# Pentamethylquercetin Regulates Lipid Metabolism by Modulating Skeletal Muscle-Adipose Tissue Crosstalk in Obese Mice

**DOI:** 10.3390/pharmaceutics14061159

**Published:** 2022-05-29

**Authors:** Jianzhao Wu, Jingxia Du, Zhi Li, Wei He, Min Wang, Manwen Jin, Lei Yang, Hui Liu

**Affiliations:** 1 Department of Pharmacology, School of Basic Medicine, Tongji Medical College, Huazhong University of Science and Technology, Wuhan 430030, China; perfectkerry@163.com (J.W.); dujingxia2005@163.com (J.D.); lizhi860131@163.com (Z.L.); hewei15152022@163.com (W.H.); wangmin5fly@163.com (M.W.); mwjin@tjmu.edu.cn (M.J.); 2 Hubei Key Laboratory of Drug Target Research and Pharmacodynamic Evaluation, Huazhong University of Science and Technology, Wuhan 430030, China; 3 Department of Anesthesiology, Union Hospital, Tongji Medical College, Huazhong University of Science and Technology, Wuhan 430022, China

**Keywords:** pentamethylquercetin, skeletal muscle, irisin, browning of white adipose tissue, AMP-activated protein kinase, peroxisome proliferator-activated receptor-γ coactivator-1α, fibronectin type III domain-containing 5

## Abstract

Irisin is an exercise-induced hormone that regulates lipid metabolism. The present study investigates whether the anti-obesity effect of the natural flavonoid pentamethylquercetin (PMQ) is related to irisin secretion from skeletal muscle in whole animals and cultured cells. Obese mice induced by monosodium glutamate were administered oral PMQ to determine blood irisin level and in vivo parameters of lipid metabolism, and cultured mouse C2C12 myoblasts and 3T3-L1 preadipocytes were employed to investigate the related molecular identities. PMQ increased circulating irisin and decreased bodyweight, insulin, and lipid levels accompanied with increasing brown-like adipocyte formation in obese mice. The brown adipocyte marker uncoupling protein 1 (UCP-1) and other brown-like adipocyte-specific genes and/or markers were increased in mouse white fat tissue, while PMQ treatment reversed the above changes. PMQ also dose-dependently increased the reduced levels of AMP-activated protein kinase (AMPK), peroxisome proliferator-activated receptor-γ coactivator-1α (PGC-1α), and fibronectin type III domain-containing 5 (FNDC5) signal molecules in obese mice. Interestingly, the irisin level was increased in the culture medium of C2C12 cells treated with PMQ, and the conditioned medium stimulated the brown-like transition of 3T3-L1 preadipocytes with the increased expression of PGC-1α, FNDC5, UCP-1, and other brown-like adipocyte-specific genes. The effects of conditioned culture medium were abolished in C2C12 cells with silenced PGC-1α. On the other hand, PMQ-induced upregulation of PGC-1α and FNDC5 expression was reduced by AMPK inhibitor Compound C in C2C12 cells. Our results demonstrate the novel information that PMQ-induced irisin secretion from skeletal muscle involves the improvement of metabolic dysfunction in obese mice via activating the AMPK/PGC-1α/FNDC5 signal pathway, suggesting that PMQ modulates skeletal muscle-adipose tissue crosstalk and may be a promising drug candidate for treating obesity and obesity-related metabolic diseases.

## 1. Introduction

The prevalence of obesity is a major public health concern worldwide [1]. Obesity results from a chronic energy imbalance [2], in which energy intake exceeds energy expenditure, which foreshadows hypertension, insulin resistance, type 2 diabetes mellitus (T2DM), and major cardiovascular diseases [3,4]. It is well recognized that adipocytes play a substantial role in the pathogenesis and complications of obesity. Two types of fat, white adipose tissue (WAT) and brown adipose tissue (BAT) are identified in mammals. WAT is specialized as an energy reservoir to store excess chemical energy in the form of triglycerides that can be released as free fatty acids into the circulation in times of food shortage [5], while BAT is responsible for dissipating chemical energy as heat to protect against hypothermia and obesity-dependent uncoupling protein 1 (UCP-1)-induced thermogenesis [6,7]. The UCP-1 is considered to be a unique biomarker specific to brown adipocytes, and uncouples respiration from ATP synthesis through catalyzing a proton leak across the inner mitochondrial membrane, thereby generating heat [8]. A distinct type of thermogenic fat cells, interspersed brown-like adipocytes (also called “beige” cells) express the markers CD137, Tbx-1, and TMEM26, which are different from classical brown adipocytes [9]. When exposed to cold [10], local hyperthermia [11], a β-adrenergic agonist or a cannabinoid receptor type 1 antagonist [12,13], and an autocrine regulator [14], beige adipocytes of the basal state or precursors appear to be morphologically and histochemically brown-like [9]. They, like classical BAT, are able to elevate fuel oxidation and thermogenesis. Experimental mice with ablation of beige adipose cells are more prone to obesity on a high-fat diet [15]. The induction of the browning of white fat might be a potential strategy for increasing whole-body energy expenditure and combating obesity [16,17].

It is well established that adipose tissue is an important contributor to the regulation of energy metabolism through organ crosstalk including crosstalk with skeletal muscle [18]. Being recognized as an endocrine organ itself, skeletal muscle secretes a panel of cytokines and proteins named myokines [19]. Irisin is a hormone-like myokine mainly secreted by skeletal muscle in response to exercise, which is proposed to induce the browning of WAT, thus increasing thermogenesis and energy expenditure [20]. Exercise activates peroxisome proliferator-activated receptor-γ coactivator-1α (PGC-1α) in muscle, and irisin, a PGC-1α-dependent myokine, is released from skeletal muscle and proteolytically cleaved from fibronectin type III domain-containing 5 (FNDC5) into the bloodstream [20]. FNDC5/irisin exerts its biological effects through several intracellular signaling pathways. The major signaling pathway involved in browning of white adipocytes is thought to be mitogen-activated protein kinase (MAPK) signaling pathways, and other essential functions of FNDC5/irisin are mediated through additional pathways including the AMP-activated protein kinase (AMPK) pathway, PI3K/AKT, and STAT3/Snail [21,22]. By binding to an α-integrin receptor [23], irisin is capable of driving brown-like adipocytes through the activation of the p38 MAPK and extracellular signal-related kinase (ERK) pathways, ameliorating obesity and glucose homeostasis in obese mice [20,24]. Irisin remains an appealing therapeutic target for metabolic diseases, albeit much research is still needed [25,26]. The pharmacological induction of irisin may be of interest to promote weight loss and improve insulin sensitivity in patients who cannot benefit from the beneficial effects of exercise [25,27]. 

Pentamethylquercetin (PMQ) is a natural polymethoxylated flavonoid. We have recently reported that PMQ possesses beneficial effects such as anti-diabetes, anti-obesity, anti-tumor, and cardioprotective properties [28,29,30,31,32], and also that PMQ can drive brown-like transition in 3T3-L1 cells as well as the transformation of WAT to brown fat in high-fat diet fed mice [33]. Studies showed that PMQ could exert actions via a number of signaling pathways, including sestrins/Keap1/Nrf2, SIRT1, TGF-β/Smads, and AMPK-related signaling pathways [30,34,35,36]. However, the underlying molecular mechanisms on how PMQ induces WAT browning are not fully understood. 

AMPK has been proved to directly phosphorylate PGC-1α and increase PGC-1α-dependent activation of its own promoter in skeletal muscle [37]. Our previous study demonstrated that PMQ could activate AMPK and elevate PGC-1α in C2C12 myotubes [30]. Therefore, it would be worth investigating further whether the AMPK-PGC-1α-FNDC5 pathway in skeletal muscle is involved in the action of PMQ, and whether PMQ regulates irisin secretion and subsequently induces browning of WAT. The present study was designed to determine whether the anti-obesity effect of PMQ is related to irisin secretion in obese mice and in ex vivo cell culture experiments. 

## 2. Materials and Methods

### 2.1. Chemicals

PMQ was synthesized by the Food & Drug Evaluation Centre of Tongji Medical College at Huazhong University of Science and Technology at a purity of 99.5% as determined by HPLC [38]. AMPK inhibitor Compound C (6-[4-(2-piperidin-1-yl-ethoxy)phenyl]-3-pyridin-4-yl-pyrazolo [1,5-a]pyrimidine), Dorsomorphin), 3-isobutyl-1-methylxanthine (IBMX), dexamethasone, and insulin were purchased from Sigma-Aldrich (St. Louis, MO, USA). Dulbecco’s modified Eagle’s medium (DMEM) and fetal bovine serum (FBS) were purchased from Gibco (Grand Island, NY, USA), and horse serum was from Hyclone (Logan, UT, USA). Radioimmunoassay kit for insulin was obtained from Beijing North Institute (F01TB, Beijing, China). ELISA kit for determination of irisin level in plasma and conditioned medium was obtained from Myhalic Biotechnology (MU30871, Wuhan, China). Lactate dehydrogenase (LDH) assay kit was from Beyotime (C0016, Shanghai, China). TRIzol reagent and Lipofectamine 2000 were purchased from Invitrogen (Carlsbad, CA, USA). Small interfering RNA (siRNA) molecules targeting PGC-1α and scrambled control siRNA were synthesized by RiboBio (Guangzhou, Guangdong, China). The anti-AMPK (#5831) and anti-pAMPK (#2531) antibodies were purchased from Cell Signaling Technology (Danvers, MA, USA). The anti-PGC-1α (sc-518025) antibody was purchased from Santa Cru Biotechnology (Santa Cruz, CA, USA). The anti-FNDC5 (ab131390) and anti-UCP-1 (ab10983) antibodies were obtained from Abcam (Cambridge, UK), whereas the antibody against GAPDH (60004-1-Ig) was from Proteintech (Wuhan, China). All other chemicals and reagents were of analytical grade.

### 2.2. Animal Experiments

CD-1 mice were purchased from Vital River (Beijing, China) at 10 weeks of age. The animals were housed in a temperature and humidity-controlled room (22 ± 2 °C, 55 ± 15%, respectively) under 12 h light/12 h dark cycles and fed with standard rodent chow for adapting for 2 weeks. Virgin female mice were mated with male mice at a ratio of 1:1. Obese mouse model was established using subcutaneous injection of monosodium glutamate (MSG) in neonatal mice at 3 g/kg body weight once daily from day 2 to day 8 after delivery as described previously [30]. At age of 5 weeks, all the MSG-treated male mice were randomized into four groups as follows: PMQ 0 mg/kg (Vehicle), PMQ 5 mg/kg, PMQ 10 mg/kg, and PMQ 20 mg/kg [30]. The age-matched normal male mice that were subcutaneously injected with normal saline served as a control group. PMQ was daily administrated by gastric gavage for 19 weeks. Control and Vehicle groups were administered an equipotent volume of vehicle. The animal body weight was monitored weekly. At age of 18 weeks, the oral glucose tolerance test (OGTT) was performed in a previously described manner [28]. The results of the OGTT were expressed as integrated area under the curve for glucose concentrations. At 24th week, body weight, body length, and waist circumference were measured, blood samples were collected for biochemical assessment including measurement of serum lipids, glucose, and insulin before the animals were sacrificed. The homeostasis model assessment-insulin resistance (HOMA-IR) was calculated to assess insulin resistance as follows: HOMA-IR = fasting glucose (mmol/L) × fasting insulin (μIU/mL)/22.5. Adipose tissues and gastrocnemius muscles of mice were dissected and stored at −80 °C for PCR and western blot analysis. The animal experiment protocol was approved by the Ethics Committee of Animal Use for Teaching and Research of Tongji Medical College, Huazhong University of Science and Technology (Wuhan, China) (permission No. S794, 2018).

### 2.3. Hematoxylin and Eosin (H&E) and Immunohistochemical Staining

The WAT tissue (inguinal fat tissue) dissected from mice were fixed in 10% formalin in phosphate buffer (pH 7.4) overnight at room temperature, embedded in paraffin, and sectioned. Tissue sections were stained with H&E or with UCP-1 primary antibody (1:600 dilution) following standard procedures.

### 2.4. Cell Culture and Treatment

Mouse C2C12 myoblast cells were purchased from Boster (Wuhan, China) and grown in DMEM containing 10% FBS, 100 U/mL penicillin, and 100 μg/mL streptomycin at 37 °C in a humidified atmosphere with 5% CO_2_. Once cells reached confluence, the cell culture medium was switched to DMEM supplemented with 2% horse serum, which was changed every 2 days. After 4 days, the C2C12 cells were considered to be myotubes [39]. Thereafter, C2C12 myotubes were incubated with various concentrations of PMQ (1, 3, 10, or 30 μM) for 0, 4, 8, 16, or 24 h. Cell viability was measured by CCK-8 test performed as described previously [30]. LDH release in the medium was detected with an LDH assay kit. To inhibit AMPK, cells were pretreated with Compound C before PMQ treatment.

Differentiated C2C12 cells were incubated with PMQ (1, 3, and 10 μM) or vehicle for 16 h. Then the cells were cultured for another 24 h without PMQ or vehicle. The resulting medium was collected, filtered, and used as conditioned medium.

Mouse 3T3-L1 preadipocytes obtained from the American Type Culture Collection (Manassas, VA, USA) were cultured in DMEM supplemented with 10% FBS, 100 U/mL penicillin, and 100 μg/mL streptomycin in 5% CO_2_ at 37 °C. Upon reaching confluence, the cells were incubated in DMEM containing 10% FBS with 0.5 mM IBMX, 0.25 μM dexamethasone and 10 μg/mL insulin for 2 days. Then, the cells were cultured for a further 2 days in DMEM supplemented with 10% FBS and 10 μg/mL insulin. Thereafter, the medium was replaced with maintenance medium (DMEM containing 10% FBS) for an additional 4 days. For the myotube conditioned medium (MCM) treatment, 3T3-L1 cells were maintained in differentiation medium containing 50% MCM for 8 days until harvest. For the FNDC5 antibody-neutralizing experiments, FNDC5 antibody at a concentration of 1.25 μg/mL was added to the MCM.

### 2.5. Reverse Transcription-Polymerase Chain Reaction (RT-PCR) Analysis

Total RNA was prepared with TRIzol reagent following the manufacturer’s protocols. RT-PCR was carried out with MyCycler PCR System (Bio-Rad, Hercules, CA, USA) as previously described [38]. The primer sequences used are listed in Supplementary Table S1.

### 2.6. Western Blot Analysis

Western blot analysis for proteins of isolated tissues and harvested cells were performed as described previously [30]. Total proteins were determined using BCA assay and then immunoblotted with primary antibodies specific for pAMPK (1:1000), AMPK (1:1000), PGC-1α (1:800), FNDC5 (1:1000), UCP-1 (1:1000), and GAPDH (1:5000).

### 2.7. siRNA Transfection

When the C2C12 cells had grown to 60–70% confluence in 6-well plate, the cells were transfected with siRNA targeting siRNA-PGC-1α or siRNA-scramble (negative control) with Lipofectamine 2000. SiRNA-PGC-1α and siRNA-scramble were synthesized and transfected according to the manufacturer’s instruction.

### 2.8. Statistical Analysis

Sample size calculation was based on our previous experiments that observed significant effects [29,30,33]. All data are expressed as means ± SEM. Statistical analysis was performed using Graph Pad Prism 5.0 software (San Diego, CA, USA). Data were analyzed for normality with the Shapiro–Wilk test. Group comparisons were evaluated by one-way ANOVA followed by Tukey’s post-hoc test. The differences were considered statistically significant at *p* < 0.05.

## 3. Results

### 3.1. PMQ Reversed Reduction of Plasma Irisin Level in Obese Mice

To investigate whether the anti-obesity effect of PMQ is related to irisin secretion, an obese mouse model was established using MSG in neonatal mice as described previously [30]. As we previously reported [30], body weight and obese indexes were greater in obese mice than in age-matched animals (Figure S1). PMQ reduced the body weight (Figure S1A), waist circumstance (Figure S1B), Lee index (Figure S1C), total fat pad weight (Figure S1D), and total fat index (Figure S1E) in a dose-dependent manner. In addition, PMQ resulted in a significant reduction of blood glucose and an improved glucose intolerance and insulin resistance in obese mice (Figure S1F–I).

**Figure 1:**
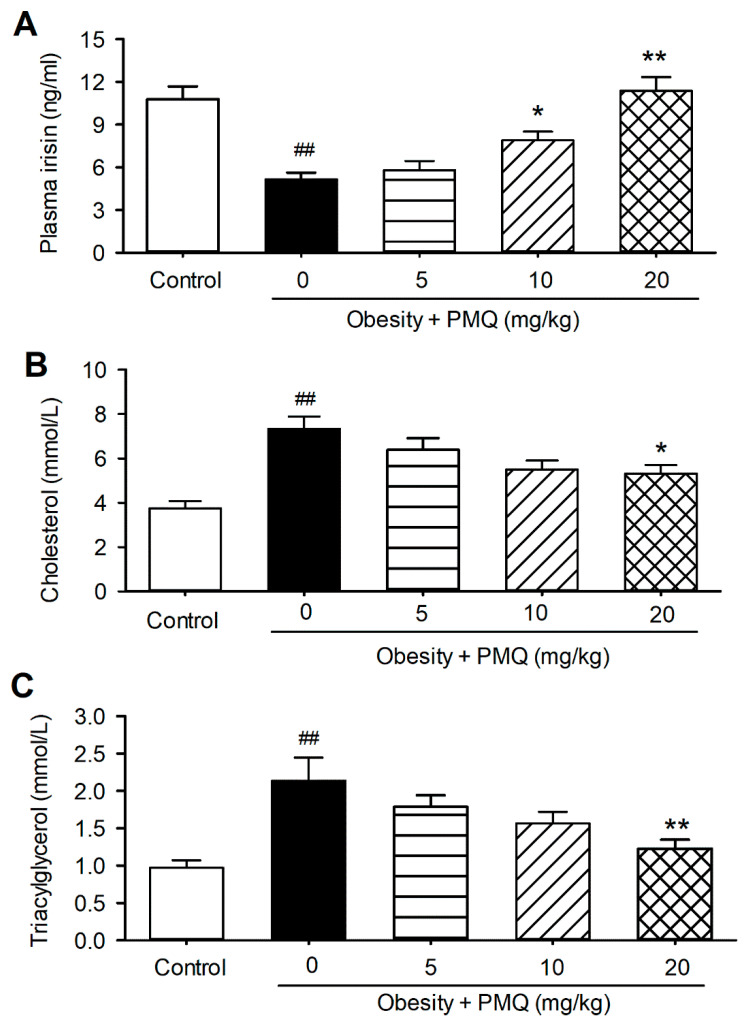
PMQ increases irisin level and regulates lipid metabolism in mice. Vehicle or PMQ was administered orally to 5-week-old normal or MSG-induced obese mice for 19 weeks. (**A**) Serum irisin levels. (**B**) total cholesterol levels. (**C**) triacylglycerol levels. ## *p* < 0.01 vs. control group; * *p* < 0.05, ** *p* < 0.01 vs. obesity group. Data are expressed as means ± SEM, *n* = 8–10.

Interestingly, plasma irisin was remarkably reduced in obese mice (5.1 ± 0.5 ng/mL vs. 10.8 ± 0.9 ng/mL of age-matched control mice, *n* = 8–10, *p* < 0.01) (Figure 1A). The decrease in plasma irisin was accompanied with an increase in plasma total cholesterol (Figure 1B) and triacylglycerol (Figure 1C) in obese mice, compared with age-matched control mice (cholesterol: 3.7 ± 0.3 mM vs. 7.3 ± 0.3 mM, *n* = 8–10, *p* < 0.01; triacylglycerol: 0.9 ± 0.1 mM vs. 2.3 ± 0.3 mM, *n* = 8–10, *p* < 0.01). PMQ reversed the reduction of irisin in a dose-dependent manner (Figure 1A). Irisin was 11.4 ± 0.9 ng/mL at 20 mg/kg (*n* = 8, *p* < 0.01 vs. obese mice). On the other hand, PMQ dose-dependently decreased the total cholesterol and triacylglycerol in obese mice (Figure 1B,C), the effect is parallel to the plasma irisin level in animals treated with PMQ. These results suggest that the beneficial effect of PMQ on lipid metabolic malfunction is likely related to irisin secretion.

### 3.2. PMQ Enhanced Skeletal Muscle PGC-1α/FNDC5 Expression in Obese Mice

**Figure 2:**
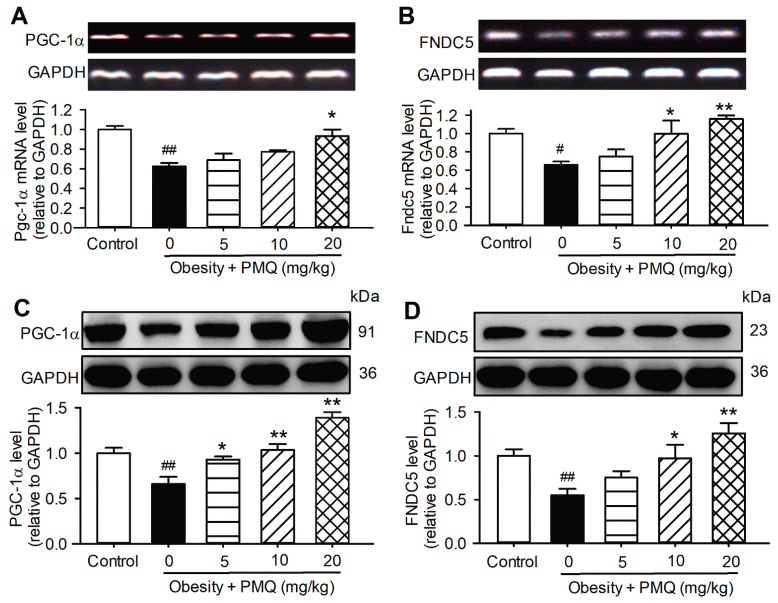
PMQ increases muscular PGC-1α and FNDC5 expression level in mice. (**A**,**B**) Relative mRNA levels of *Pgc*-*1α* and *Fndc5* in gastrocnemius muscle of mice analyzed by RT-PCR. *n* = 3. (**C**,**D**) Protein levels of PGC-1α and FNDC5 in gastrocnemius muscle of mice examined by western blot. *n* = 4. *# p* < 0.05, ## *p* < 0.01 vs. control group; * *p* < 0.05, ** *p* < 0.01 vs. obesity group. Data are expressed as means ± SEM.

It has been recognized that irisin is a cleaved version of FNDC5 and secreted from skeletal muscle. Exercise causes an increased expression of PGC-1α in skeletal muscle, then increases FNDC5 expression to give a new product, irisin [20]. We therefore examined the mRNA and protein expression of PGC-1α and FNDC5 in the skeletal muscle of obese mice treated with PMQ. The mRNA and protein levels of PGC-1α and FNDC5 were significantly decreased in the skeletal muscle of obese mice, compared with age-matched control mice (Figure 2). The reduced PGC-1α and FNDC5 were upregulated in mice treated with 5 mg/kg, 10 mg/kg, and 20 mg/kg PMQ. These results suggest that PMQ may increase PGC-1α and FNDC5 expression by mimicking exercise and skeletal muscle contraction to secrete irisin into circulation.

### 3.3. PMQ Promoted Browning of WAT in Obese Mice

**Figure 3:**
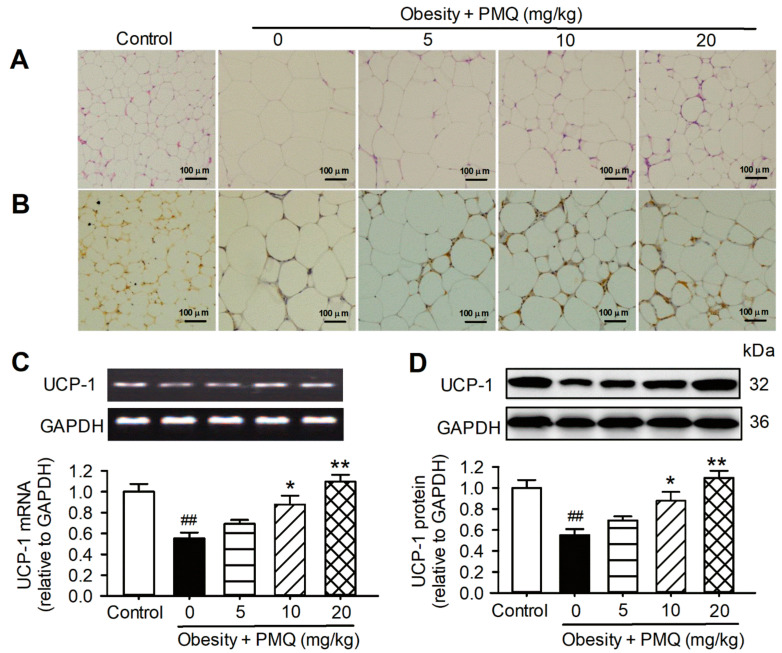
PMQ promotes the browning of inguinal WAT in MSG mice. (**A**) Representative images of H&E-stained paraffin sections of inguinal fat in the groups indicated (×100). (**B**) Representative images from UCP-1 immunohistochemistry on sections of inguinal fat (×100). (**C**) Relative mRNA levels of UCP-1 in inguinal WAT. (**D**) UCP-1 protein levels in inguinal WAT. ## *p* < 0.01 vs. control group; * *p* < 0.05, ** *p* < 0.01 vs. obesity group. Data are expressed as means ± SEM, *n* = 4.

It has been proposed that irisin promotes the conversion of white fat to beige fat in mammals [24]. We therefore analyzed histologic and molecular alterations of WAT in obese mice treated with PMQ. H&E staining revealed that the size of adipocytes was larger in obese mice than in the age-matched control mice. PMQ treatment decreased the enlarged cell size in WAT in a dose-dependent manner (Figure 3A). Immunohistological staining with antibodies of the brown fat cell marker UCP-1 showed a gradual decrease in cell size and an increase in cell number with a stained brown color with increasing PMQ dosage (Figure 3B). The mRNA (Figure 3C) and protein (Figure 3D) levels of UCP-1 were remarkably decreased in the WAT of obese mice and upregulated in obese mice treated with PMQ. These results suggest that brown-like adipocyte formation and the upregulation of UCP-1 may be related to irisin secretion in obese mice treated with PMQ.

In addition, mRNA levels of other brown-specific genes (Pgc-1α, Cidea, and Cox7a1) and beige cell markers (Tbx1, Tmem26, and CD137) were decreased in the WAT of obese mice. PMQ treatment reversed the reduced mRNA expression (Figure S2), which further supports the observation that PMQ increases irisin secretion from skeletal muscle and improves the impaired lipid metabolism in obese mice.

### 3.4. PMQ Increased Irisin Secretion in Cultured C2C12 Myotubes

**Figure 4:**
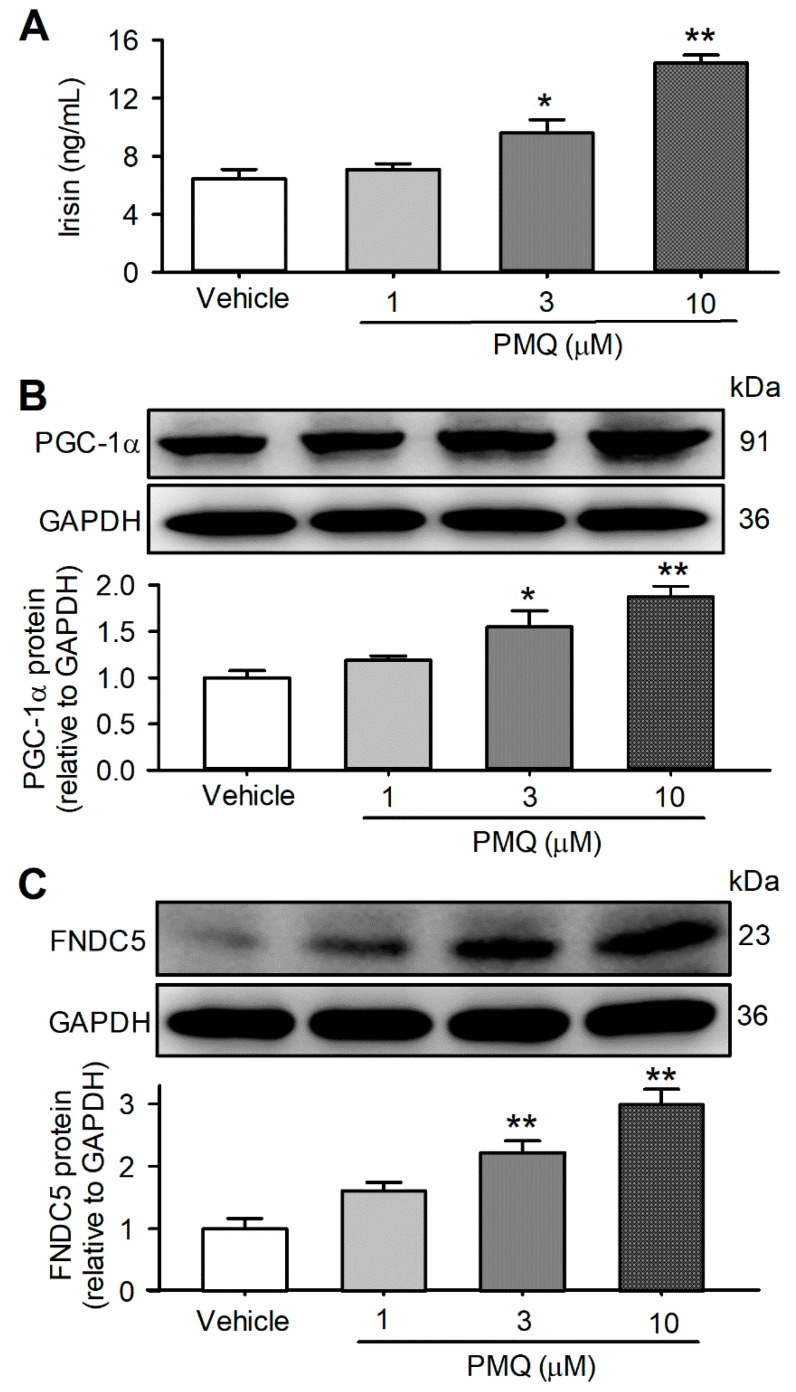
PMQ increases irisin secretion and upregulates the expression of PGC-1α and FNDC5 in C2C12 myotubes. C2C12 myotubes were treated with various concentrations of PMQ (1, 3, 10 μM) or vehicle for 16 h, and cultured for an additional 24 h in regular DMEM. Then the conditioned media were collected. (**A**) Irisin levels in the conditioned medium. *n* = 6. (**B**,**C**) Protein levels of PGC-1α and FNDC5 in C2C12 myotubes. *n* = 3. * *p* < 0.05, ** *p* < 0.01 vs. vehicle. Data are expressed as means ± SEM.

To investigate whether a PMQ-induced increase in irisin could be observed in cultured cells, we determined the irisin level in the culture medium of differentiated C2C12 cells treated with 1, 3, and 10 μM PMQ for 16 h, and then in regular DMEM for 24 h. Interestingly, irisin was increased by PMQ in a concentration-dependent manner (Figure 4A). Irisin was 6.5 ± 0.6 ng/mL in the vehicle control medium, 7.1 ± 0.4 ng/mL, 9.6 ± 0.9 ng/mL (*p* < 0.05 vs. control), and 14.4 ± 0.6 ng/mL (*n* = 6, *p* < 0.01 vs. control), respectively, in the culture medium of cells treated by 1, 3, and 10 μM PMQ. PGC-1α and FNDC5 protein expression were increased by 1, 3, and 10 μM PMQ in a concentration-dependent manner (Figure 4B,C), and the efficacy was parallel to the irisin level of the culture medium, which implicates that irisin secretion is correlated to the increased expression of PGC-1α and FNDC5 proteins in C2C12 myotubes.

### 3.5. PMQ Promoted Browning Transition Markers in 3T3-L1 Cells by FNDC5/Irisin Pathway

**Figure 5:**
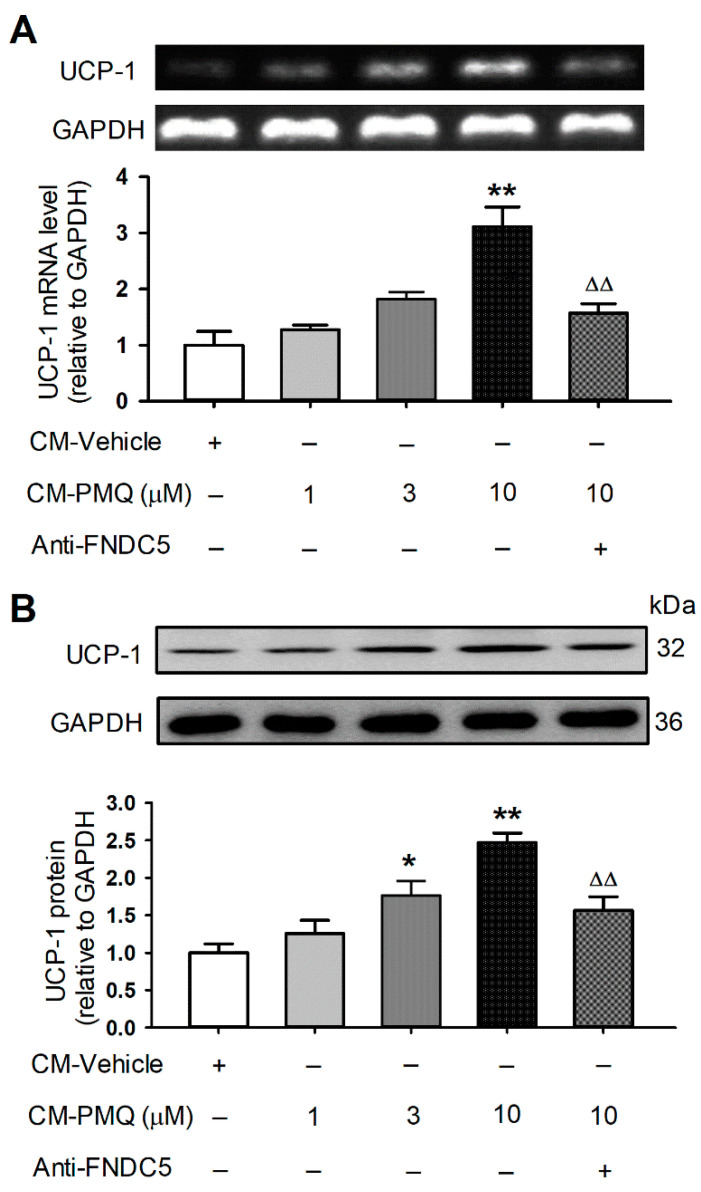
PMQ promotes UCP-1 expression in 3T3-L1 cells by FNDC5/irisin pathway. 3T3-L1 cells were treated with CM-vehicle, CM-PMQ (1, 3, 10) and CM-PMQ10 plus FNDC5 antibody (anti-FNDC5). (**A**) Relative mRNA levels of UCP-1 in 3T3-L1 cells. *n* = 3. (**B**) UCP-1 protein levels in 3T3-L1 cells. *n* = 4. * *p* < 0.05, ** *p* < 0.01 vs CM-vehicle; ΔΔ *p* < 0.01 vs. CM-PMQ10. Data are expressed as means ± SEM.

If FNDC5/irisin is responsible for the browning of WAT adipocytes by PMQ in obese mice, the culture medium from differentiated C2C12 cells may also have such an effect. To test if it is the case, we collected the conditioned culture medium of C2C12 myotube cells treated with PMQ, then used the conditioned medium to culture 3T3-L1 preadipocytes (Figure S3). Interestingly, the mRNA and protein levels of UCP-1 (a typical browning adipocyte marker) were increased in 3T3-L1 preadipocytes cultured with the conditioned medium of C2C12 cells treated with 1, 3, and 10 μM PMQ. The effect was reduced by anti-FNDC5 antibody incubation (Figure 5). In addition, other brown adipocyte signature genes (Pgc-1α, Cidea, and Cox7a1) and beige-cell marker genes (Cd137, Tbx1, and Tmem26) were also increased in 3T3-L1 preadipocytes cultured with conditioned medium of C2C12 myotube cells treated with 1, 3, and 10 μM PMQ, and the effect was decreased in cells incubated with anti-FNDC5 antibody (Figure S4). These results support the notion that the browning transition of adipocytes induced by PMQ is mediated by FNDC5/irisin.

### 3.6. PMQ Induced White Adipocyte Browning via Upregulating PGC-1α

**Figure 6:**
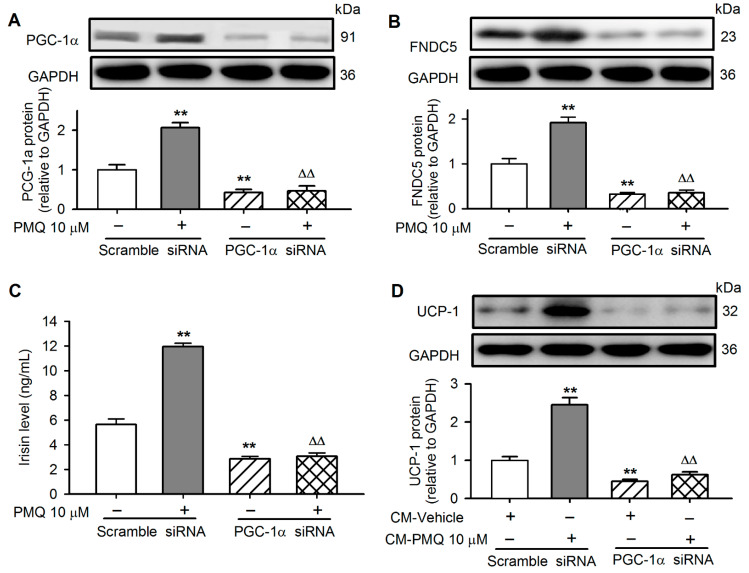
Down-regulation of PGC-1α blocks the stimulatory effects of conditioned medium from PMQ-treated myotubes on UCP-1 expression in 3T3-L1 adipocytes. (**A**,**B**) C2C12 cells were transfected with siRNA-scramble or siRNA-PGC-1α, exposed to DMSO or PMQ 10 μM for 16 h, the protein levels of PGC-1α and FNDC5 in C2C12 cells were quantified by western blot. (**C**) After C2C12 cells were transfected with siRNA-scramble or siRNA-PGC-1α and PMQ incubation, and then cultured with regular DMEM for an additional 24 h, the irisin concentration in CM was detected. (**D**) UCP-1 protein levels in 3T3-L1 cells incubated with conditioned medium from C2C12 cells. ** *p* < 0.01 vs. scramble siRNA without PMQ; ΔΔ *p* < 0.01 vs. scramble siRNA with PMQ. Data are expressed as means ± SEM. *n* = 3–6.

PGC-1α was silenced in C2C12 cells transfected with siRNA targeting PGC-1α to investigate whether PGC-1α plays a role in mediating PMQ effect on browning adipocytes. The silencing of PGC-1α remarkably decreased its protein level and abolished the PMQ-induced upregulation (Figure 6A), and also reduced FNDC5 expression and PMQ-induced upregulation (Figure 6B), indicating that FNDC5 expression is dependent on PGC-1α. The decreased FNDC5 expression resulted in the reduction of irisin secretion. Irisin in the cultured medium was 5.6 ± 0.4 ng/mL in cells with scramble siRNA, 2.9 ± 0.4 ng/mL in cells with PGC-1α siRNA (*n* = 6, *p* < 0.01), and 11.9 ± 0.3 ng/mL in cells with scramble siRNA and 10 μM PMQ, while it was 3.1 ± 0.3 ng/mL in cells with PGC-1α siRNA and 10 μM PMQ, *n* = 6, *p* < 0.01) (Figure 6C). It is interesting to note that UCP-1 protein was reduced and no longer upregulated by PMQ in 3T3-L1 preadipocytes cultured with conditioned medium of C2C12 cells transfected with PGC-1α siRNA (Figure 6D), in which irisin was remarkably reduced (Figure 6C). Taken together, these results indicate that the PMQ-induced browning transition of adipocytes is mediated by activating PGC-1α/FNDC5/irisin.

### 3.7. PMQ Upregulated PGC-1α-FNDC5 Pathway by Activating AMPK

**Figure 7:**
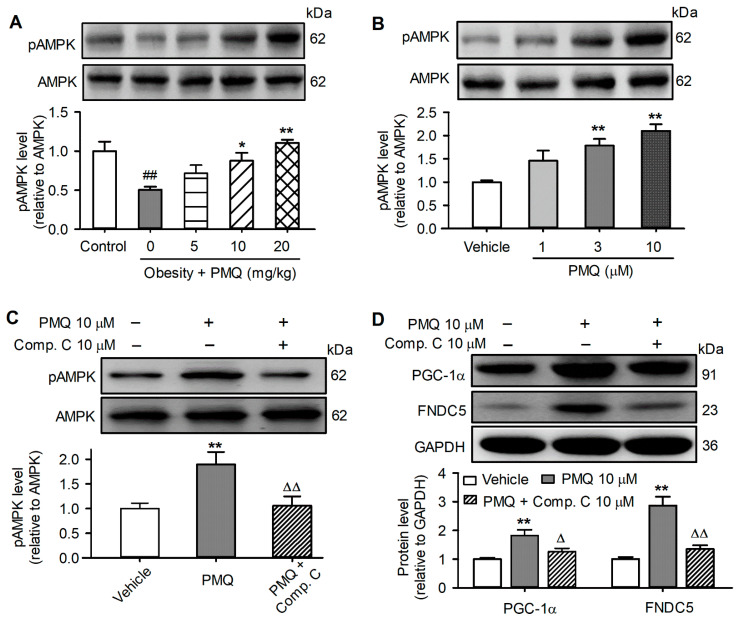
PMQ enhances PGC-1α and FNDC5 protein expression by activating AMPK. (**A**) Protein levels of pAMPK and AMPK in gastrocnemius muscle of mice. ## *p* < 0.01 vs. control group; * *p* < 0.05, ** *p* < 0.01 vs. MSG group. (**B**) Protein levels of pAMPK and AMPK in C2C12 myotubes treated with various concentrations of PMQ (1, 3, 10 μM) or vehicle for 16 h. ** *p* < 0.01 vs. vehicle. (**C**,**D**) Protein levels of pAMPK, AMPK, PGC-1α, and FNDC5 in C2C12 Myotubes pretreated with or without 10 μM compound C for 6 h and continuously incubated with PMQ for 16 h. ** *p* < 0.01 vs. vehicle; Δ *p* < 0.05, ΔΔ *p* < 0.01 vs. PMQ 10. Data are expressed as means ± SEM, *n* = 4.

As we previously reported [30], PMQ increased the AMPK phosphorylation level in the skeletal muscle of obese mice (Figure 7A) and in C2C12 cells (Figure 7B). The AMPK inhibitor Compound C almost fully prevented pAMPK upregulation by PMQ (Figure 7C). If AMPK mediates the activation of PGC-1α/FNDC5, Compound C would prevent the PMQ-induced increase in these two proteins. Indeed, Compound C (10 μM) remarkably decreased PMQ-induced upregulation of PGC-1α and FNDC5 in C2C12 cells (Figure 7D). These results suggest that the lipid metabolism regulation by PMQ is mediated by the PGC-1α/FNDC5/irisin pathway at least in part via activating AMPK.

## 4. Discussion

The global obesity epidemic continues its relentless advance, currently affecting more than 2 billion people [40]. It is generally believed that the promotion of energy-storing white adipocytes into heat-producing beige adipocytes in WAT is a promising strategy for fighting against obesity and its complications. In our experiments, PMQ treatment resulted in a decreased adipocyte size with an appearance of multilocular lipid droplets of WAT in MSG-induced obese mice, which is the same as we previously reported in high-fat food-diet-induced obese mice [33]. mRNAs of brown adipocytes such as Ucp-1, Pgc-1α, Cidea, and Cox7a1 as well as the UCP-1 protein were upregulated by PMQ treatment. Meanwhile, the mRNA levels of beige adipocyte markers such as Tbx1, Tmem26, and Cd137 were increased in obese mice treated with PMQ. These data strongly support the notion that PMQ promotes the brown-like adipocyte formation in WAT of obese mice.

Skeletal muscle has been recognized to work as an endocrine organ, and the crosstalk between skeletal muscle and adipose tissue plays an important role in the metabolic homeostasis of the whole body [41,42]. Irisin is an exercise-induced myokine, it is a protein product of the FNDC5 gene that encodes the type I membrane protein, the extracellular domain of which can be proteolysed to form irisin in skeletal muscle [20]. Clinical studies found that circulating irisin levels were correlated reversely with body weight [43], hepatic triglyceride [44], insulin sensitivity [45], urea nitrogen, and creatinine [46]. Plasma irisin levels and muscular FNDC5 expression levels were reduced in patients with obesity and T2DM [47,48]. Moreover, there was an association between serum irisin and diabetic nephropathy in patients with T2DM [49]. Thus, irisin has been linked to favorable effects on metabolic diseases, such as obesity and T2DM [25]. As indicated in this study, MSG-induced obese mice had significant obesity, hyperglycaemia, hyperlipidaemia, hyperinsulinaemia, and insulin resistance. However, the plasma irisin level and FNDC5 mRNA/protein expression of skeletal muscle were significantly decreased in obese mice compared to the control animals. Therefore, we believe that irisin may play a role in lowering body weight and improving metabolic disorders.

Exercise increases FNDC5 expression in skeletal muscle and subsequently secretes irisin into circulation followed by promoting the browning of white adipocytes and improving insulin sensitivity [20,24]. Small molecule compounds or drugs that increase FNDC5 expression and circulating irisin levels are probably able to mimic the effects of exercise [27]. It has been reported that metformin, icariin, and myricanol upregulated intramuscular FNDC5 expression and stimulated irisin secretion from skeletal muscle into the blood [50,51,52,53]. In the present study, we found that PMQ restored the impaired FNDC5 of skeletal muscle and increased irisin secretion to the blood in obese mice. Similarly, FNDC5 was upregulated in cultured C2C12 myotubes by PMQ and the irisin concentration was increased in the culture medium. These results indicate that PMQ may be an FNDC5 activator that mimics the effects of exercise on FNDC5 expression. Recent studies showed that the overexpression of irisin by adenoviral vectors or the administration of recombinant irisin decreased body weight, increased brown fat-specific genes expression in subcutaneous WAT, and improved glucose intolerance in high fat diet-fed mice [20,24]. Our results showed that the conditioned medium with increased irisin level from cultured C2C12 myoblast treated with PMQ promoted the typical brown adipocyte marker UCP-1 expression, other brown adipocyte genes (Pgc-1α, Cidea, and Cox7a1), and beige cell markers (Tbx-1, Tmem26, and Cd137) in 3T3-L1 preadipocytes. Moreover, the anti-FNDC5 antibody significantly decreased the browning effect of 3T3-L1 preadipocytes by the conditioned medium of C2C12 cells treated with PMQ. Collectively, these results suggested that FNDC5/irisin plays a pivotal role in the PMQ-induced browning of white adipocytes.

It is well recognized that skeletal muscle FNDC5 expression and irisin secretion are activated by PGC-1α [20]. In skeletal muscle and C2C12 myotubes, PGC-1α expression was increased by PMQ. Silencing PGC-1α reduced FNDC5 expression, the PMQ effect, as well as irisin secretion, and also abolished the conditioned medium-induced browning transition of 3T3-L1 preadipocytes. The results further support the concept that PGC-1α activation by PMQ in skeletal muscle is critical for promoting white adipocyte browning by stimulating FNDC5/irisin.

A recent study showed that quercetin promotes white adipocytes to brown-like adipocytes in part by the activation of AMPK [54]. We have also observed that the effects of PMQ are at least partially through activating AMPK [30]. AMPK plays a major role in regulating the cellular energy balance and has attracted widespread interest as a potential therapeutic target for metabolic diseases [55]. AMPK participates in regulating the PGC-1α/FNDC5 signaling cascade [56]. PMQ significantly increased the pAMPK level in the skeletal muscle of MSG-induced obese mice [30]. The present study further confirmed that PMQ activated AMPK in the skeletal muscle of MSG-induced obese mice and in C2C12 myotubes. The AMPK inhibitor Compound C blunted PMQ-induced activation of PGC-1α and FNDC5 in C2C12 myotubes. These results imply that AMPK activation is necessary to mediate the effect of PMQ on PGC-1α/FNDC5, and the subsequent irisin secretion in skeletal muscle.

Flavonoids have been shown to induce WAT browning and activate BAT to increase energy consumption and non-shivering thermogenesis and may foster a relatively safe and effective therapeutic option to improve metabolic health [57,58]. However, factors such as a low bioavailability, a poor flavonoid stability and solubility, and ineffective targeted delivery, hinder the application of flavonoids [59]. For example, quercetin, a widely-studied natural flavonoid, has been reported to remodel white adipocytes to brown-like adipocytes [54]. Quercetin has other positive health benefits such as anti-cancer and anti-diabetic effects [60,61]. However, the application of quercetin in the pharmaceutical field is limited due to its poor solubility, low bioavailability, and poor permeability [62]. Methylated flavonoids have improved bioavailability compared with flavonoid precursors [63]. We did find that PMQ, the methylated derivative of quercetin, showed a high oral absorption rate in beagle dogs [64] and had similar biological effects to quercetin [30,32,33]; therefore, PMQ may be a more promising drug candidate. It is worth mentioning that nanoconjugated quercetin in targeting cancer has attracted much attention recently [65], nanocarriers such as stimuli-responsive nanocarriers for quercetin delivery may improve the therapeutic efficacy [66,67]. Modern delivery using nano technology would also provide a new approach for the application of PMQ.

There are some limitations present in this study. One of them was that only the levels of total cholesterol and triacylglycerol were determined; whether PMQ affects low-density lipoprotein cholesterol (LDL-C) or high-density lipoprotein cholesterol (HDL-C) was not studied. We previously reported that PMQ administration resulted in significant reductions in serum lipids including LDL-C levels in high-fat diet-fed mice [33]. Considering that not only a high level of LDL-C, but also a low level of HDL-C, is a critical risk factor for atherosclerosis, it would be worthwhile to investigate if PMQ could affect HDL-C levels and improve the atherogenic index of plasma. Another limitation was the lack of PMQ treatment in normal mice, in which may reveal a potential toxicity of PMQ. However, these limitations would not affect the conclusion of this study.

## 5. Conclusions

**Figure 8:**
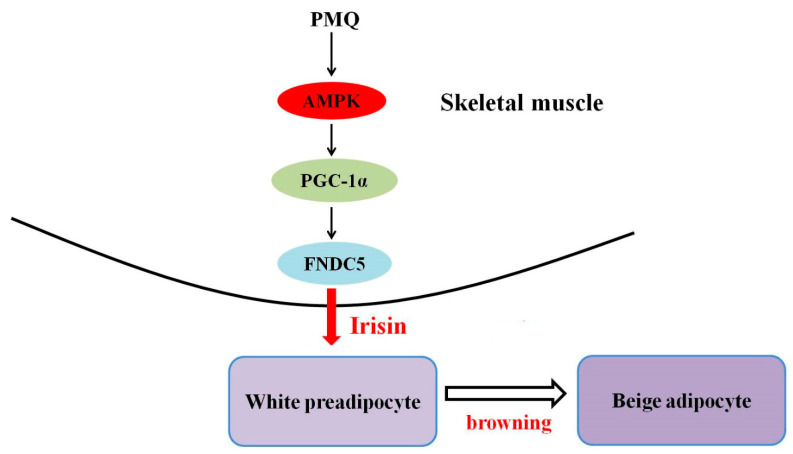
Schematic diagram of molecular mechanism of PMQ on driving browning of WAT.

Collectively, our in vivo and in vitro data show that PMQ regulated lipid metabolism and induced the browning of WAT. PMQ also exerted beneficial effects against obesity, hyperglycaemia, hyperinsulinaemia, and insulin resistance in obese mice. The findings of the present study demonstrate the novel information that the anti-obesity effect of PMQ is related to an increased irisin secretion from skeletal muscle, thereby promoting brown-like adipocytes formation in WAT, at least in part via activating the AMPK/PGC-1α/FNDC5 pathway (Figure 8), which mimics the effects of exercise. Our results suggest that PMQ modulates skeletal muscle-adipose tissue crosstalk and may be a promising drug candidate for treating obesity and obesity-related metabolic disorders. It will be of future interest to clarify whether other metabolism-related signaling pathways are involved in the effects of PMQ.

## Data Availability

Data are contained within the article.

## Supplementary Materials

The following supporting information can be downloaded at: https://www.mdpi.com/article/10.3390/pharmaceutics14061159/s1, Figure S1: PMQ reduces obesity and improves insulin sensitivity.; Figure S2: PMQ promotes the browning of inguinal WAT in MSG mice; Figure S3: Experimental scheme for myotube-conditioned media treatment on 3T3-L1 adipocytes; Figure S4: Conditioned media from PMQ-treated myotubes induce brown-like transition in 3T3-L1 cells; Table S1: primer sequence.

## References

[1] NCD Risk Factor Collaboration (2016). Trends in adult body-mass index in 200 countries from 1975 to 2014: A pooled analysis of 1698 population-based measurement studies with 19.2 million participants. Lancet.

[2] Spiegelman B.M., Flier J.S. (2001). Obesity and the regulation of energy balance. Cell.

[3] Kahn S.E., Hull R.L., Utzschneider K.M. (2006). Mechanisms linking obesity to insulin resistance and type 2 diabetes. Nature.

[4] Lavie C.J., Milani R.V., Ventura H.O. (2009). Obesity and cardiovascular disease: Risk factor, paradox, and impact of weight loss. J. Am. Coll. Cardiol..

[5] Attie A.D., Scherer P.E. (2009). Adipocyte metabolism and obesity. J. Lipid. Res..

[6] Cannon B., Nedergaard J. (2004). Brown adipose tissue: Function and physiological significance. Physiol. Rev..

[7] Enerback S. (2010). Human brown adipose tissue. Cell Metab..

[8] Nedergaard J., Ricquier D., Kozak L.P. (2005). Uncoupling proteins: Current status and therapeutic prospects. EMBO Rep..

[9] Cohen P., Kajimura S. (2021). The cellular and functional complexity of thermogenic fat. Nat. Rev. Mol. Cell Biol..

[10] Shan B., Shao M., Zhang Q., An Y.A., Vishvanath L., Gupta R.K. (2021). Cold-responsive adipocyte progenitors couple adrenergic signaling to immune cell activation to promote beige adipocyte accrual. Genes Dev..

[11] Li Y., Wang D., Ping X., Zhang Y., Zhang T., Wang L., Jin L., Zhao W., Guo M., Shen F. (2022). Local hyperthermia therapy induces browning of white fat and treats obesity. Cell.

[12] Jiang Y., Berry D.C., Graff J.M. (2017). Distinct cellular and molecular mechanisms for beta3 adrenergic receptor-induced beige adipocyte formation. eLife.

[13] Behl T., Chadha S., Sachdeva M., Sehgal A., Kumar A., Dhruv, Venkatachalam T., Hafeez A., Aleya L., Arora S. (2021). Understanding the possible role of endocannabinoid system in obesity. Prostaglandins Other Lipid Mediat..

[14] Abu-Odeh M., Zhang Y., Reilly S.M., Ebadat N., Keinan O., Valentine J.M., Hafezi-Bakhtiari M., Ashayer H., Mamoun L., Zhou X. (2021). FGF21 promotes thermogenic gene expression as an autocrine factor in adipocytes. Cell Rep..

[15] Cohen P., Levy J.D., Zhang Y., Frontini A., Kolodin D.P., Svensson K.J., Lo J.C., Zeng X., Ye L., Khandekar M.J. (2014). Ablation of PRDM16 and beige adipose causes metabolic dysfunction and a subcutaneous to visceral fat switch. Cell.

[16] Carey A.L., Kingwell B.A. (2013). Brown adipose tissue in humans: Therapeutic potential to combat obesity. Pharmacol. Ther..

[17] Montanari T., Poscic N., Colitti M. (2017). Factors involved in white-to-brown adipose tissue conversion and in thermogenesis: A review. Obes. Rev..

[18] Scheele C., Wolfrum C. (2020). Brown Adipose Crosstalk in Tissue Plasticity and Human Metabolism. Endocr. Rev..

[19] Gomarasca M., Banfi G., Lombardi G. (2020). Myokines: The endocrine coupling of skeletal muscle and bone. Adv. Clin. Chem..

[20] Bostrom P., Wu J., Jedrychowski M.P., Korde A., Ye L., Lo J.C., Rasbach K.A., Bostrom E.A., Choi J.H., Long J.Z. (2012). A PGC1-alpha-dependent myokine that drives brown-fat-like development of white fat and thermogenesis. Nature.

[21] Rabiee F., Lachinani L., Ghaedi S., Nasr-Esfahani M.H., Megraw T.L., Ghaedi K. (2020). New insights into the cellular activities of Fndc5/Irisin and its signaling pathways. Cell Biosci..

[22] Vliora M., Grillo E., Corsini M., Ravelli C., Nintou E., Karligiotou E., Flouris A.D., Mitola S. (2022). Irisin regulates thermogenesis and lipolysis in 3T3-L1 adipocytes. Biochim. Biophys. Acta Gen. Subj..

[23] Kim H., Wrann C.D., Jedrychowski M., Vidoni S., Kitase Y., Nagano K., Zhou C., Chou J., Parkman V.A., Novick S.J. (2018). Irisin Mediates Effects on Bone and Fat via alphaV Integrin Receptors. Cell.

[24] Zhang Y., Li R., Meng Y., Li S., Donelan W., Zhao Y., Qi L., Zhang M., Wang X., Cui T. (2014). Irisin stimulates browning of white adipocytes through mitogen-activated protein kinase p38 MAP kinase and ERK MAP kinase signaling. Diabetes.

[25] Polyzos S.A., Anastasilakis A.D., Efstathiadou Z.A., Makras P., Perakakis N., Kountouras J., Mantzoros C.S. (2018). Irisin in metabolic diseases. Endocrine.

[26] Waseem R., Shamsi A., Mohammad T., Hassan M.I., Kazim S.N., Chaudhary A.A., Rudayni H.A., Al-Zharani M., Ahmad F., Islam A. (2022). FNDC5/Irisin: Physiology and Pathophysiology. Molecules.

[27] Villarroya F. (2012). Irisin, turning up the heat. Cell Metab..

[28] Wang Y., Xin X., Jin Z., Hu Y., Li X., Wu J., Jin M. (2011). Anti-diabetic effects of pentamethylquercetin in neonatally streptozotocin-induced diabetic rats. Eur. J. Pharmacol..

[29] He T., Chen L., Chen Y., Han Y., Yang W.Q., Jin M.W. (2012). In vivo and in vitro protective effects of pentamethylquercetin on cardiac hypertrophy. Cardiovasc. Drugs Ther..

[30] Shen J.Z., Ma L.N., Han Y., Liu J.X., Yang W.Q., Chen L., Liu Y., Hu Y., Jin M.W. (2012). Pentamethylquercetin generates beneficial effects in monosodium glutamate-induced obese mice and C2C12 myotubes by activating AMP-activated protein kinase. Diabetologia.

[31] Du J.X., Wu J.Z., Li Z., Zhang C., Shi M.T., Zhao J., Jin M.W., Liu H. (2019). Pentamethylquercetin protects against cardiac remodeling via activation of Sestrin2. Biochem. Biophys. Res. Commun..

[32] Li Z., Gao W.Q., Wang P., Wang T.Q., Xu W.C., Zhu X.Y., Liu H. (2020). Pentamethylquercetin Inhibits Hepatocellular Carcinoma Progression and Adipocytes-induced PD-L1 Expression via IFN-gamma Signaling. Curr. Cancer Drug Targets.

[33] Han Y., Wu J.Z., Shen J.Z., Chen L., He T., Jin M.W., Liu H. (2017). Pentamethylquercetin induces adipose browning and exerts beneficial effects in 3T3-L1 adipocytes and high-fat diet-fed mice. Sci. Rep..

[34] Ying H.Z., Zang J.N., Deng L.L., Wang Z.Y., Yu C.H. (2013). Pentamethylquercetin reduces fat deposition via Sirt1-mediated pathways in male obese mice induced by a high fat diet. Food Chem. Toxicol..

[35] Xin X., Li X.H., Wu J.Z., Chen K.H., Liu Y., Nie C.J., Hu Y., Jin M.W. (2013). Pentamethylquercetin ameliorates fibrosis in diabetic Goto-Kakizaki rat kidneys and mesangial cells with suppression of TGF-beta/Smads signaling. Eur. J. Pharmacol..

[36] Du J., He W., Zhang C., Wu J., Li Z., Wang M., Feng S., Liang G. (2020). Pentamethylquercetin Attenuates Cardiac Remodeling via Activation of the Sestrins/Keap1/Nrf2 Pathway in MSG-Induced Obese Mice. BioMed Res. Int..

[37] Jager S., Handschin C., St-Pierre J., Spiegelman B.M. (2007). AMP-activated protein kinase (AMPK) action in skeletal muscle via direct phosphorylation of PGC-1alpha. Proc. Natl. Acad. Sci. USA.

[38] Chen L., He T., Han Y., Sheng J.Z., Jin S., Jin M.W. (2011). Pentamethylquercetin improves adiponectin expression in differentiated 3T3-L1 cells via a mechanism that implicates PPARgamma together with TNF-alpha and IL-6. Molecules.

[39] Canto C., Gerhart-Hines Z., Feige J.N., Lagouge M., Noriega L., Milne J.C., Elliott P.J., Puigserver P., Auwerx J. (2009). AMPK regulates energy expenditure by modulating NAD+ metabolism and SIRT1 activity. Nature.

[40] Caballero B. (2019). Humans against Obesity: Who Will Win?. Adv. Nutr..

[41] Li F., Li Y., Duan Y., Hu C.A., Tang Y., Yin Y. (2017). Myokines and adipokines: Involvement in the crosstalk between skeletal muscle and adipose tissue. Cytokine Growth Factor Rev..

[42] Severinsen M.C.K., Pedersen B.K. (2020). Muscle-Organ Crosstalk: The Emerging Roles of Myokines. Endocr. Rev..

[43] Crujeiras A.B., Pardo M., Arturo R.R., Navas-Carretero S., Zulet M.A., Martinez J.A., Casanueva F.F. (2014). Longitudinal variation of circulating irisin after an energy restriction-induced weight loss and following weight regain in obese men and women. Am. J. Hum. Biol..

[44] Zhang H.J., Zhang X.F., Ma Z.M., Pan L.L., Chen Z., Han H.W., Han C.K., Zhuang X.J., Lu Y., Li X.J. (2013). Irisin is inversely associated with intrahepatic triglyceride contents in obese adults. J. Hepatol..

[45] Sesti G., Andreozzi F., Fiorentino T.V., Mannino G.C., Sciacqua A., Marini M.A., Perticone F. (2014). High circulating irisin levels are associated with insulin resistance and vascular atherosclerosis in a cohort of nondiabetic adult subjects. Acta Diabetol..

[46] Wen M.S., Wang C.Y., Lin S.L., Hung K.C. (2013). Decrease in irisin in patients with chronic kidney disease. PLoS ONE.

[47] Moreno-Navarrete J.M., Ortega F., Serrano M., Guerra E., Pardo G., Tinahones F., Ricart W., Fernandez-Real J.M. (2013). Irisin is expressed and produced by human muscle and adipose tissue in association with obesity and insulin resistance. J. Clin. Endocrinol. Metab..

[48] Song R., Zhao X., Zhang D.Q., Wang R., Feng Y. (2021). Lower levels of irisin in patients with type 2 diabetes mellitus: A meta-analysis. Diabetes Res. Clin. Pract..

[49] Wang R., Liu H. (2021). Association Between Serum Irisin and Diabetic Nephropathy in Patients with Type 2 Diabetes Mellitus: A Meta-Analysis. Horm. Metab. Res..

[50] Yang Z., Chen X., Chen Y., Zhao Q. (2015). PGC-1 mediates the regulation of metformin in muscle irisin expression and function. Am. J. Transl. Res..

[51] Li D.J., Huang F., Lu W.J., Jiang G.J., Deng Y.P., Shen F.M. (2015). Metformin promotes irisin release from murine skeletal muscle independently of AMP-activated protein kinase activation. Acta Physiol..

[52] Shen S., Liao Q., Zhang T., Pan R., Lin L. (2019). Myricanol modulates skeletal muscle-adipose tissue crosstalk to alleviate high-fat diet-induced obesity and insulin resistance. Br. J. Pharmacol..

[53] Chen S.Q., Ding L.N., Zeng N.X., Liu H.M., Zheng S.H., Xu J.W., Li R.M. (2019). Icariin induces irisin/FNDC5 expression in C2C12 cells via the AMPK pathway. Biomed. Pharmacother..

[54] Lee S.G., Parks J.S., Kang H.W. (2017). Quercetin, a functional compound of onion peel, remodels white adipocytes to brown-like adipocytes. J. Nutr. Biochem..

[55] Carling D. (2017). AMPK signalling in health and disease. Curr. Opin. Cell Biol..

[56] Shan T., Liang X., Bi P., Kuang S. (2013). Myostatin knockout drives browning of white adipose tissue through activating the AMPK-PGC1alpha-Fndc5 pathway in muscle. FASEB J..

[57] Zhang X., Li X., Fang H., Guo F., Li F., Chen A., Huang S. (2019). Flavonoids as inducers of white adipose tissue browning and thermogenesis: Signalling pathways and molecular triggers. Nutr. Metab..

[58] Silvester A.J., Aseer K.R., Yun J.W. (2019). Dietary polyphenols and their roles in fat browning. J. Nutr. Biochem..

[59] Vazhappilly C.G., Amararathna M., Cyril A.C., Linger R., Matar R., Merheb M., Ramadan W.S., Radhakrishnan R., Rupasinghe H.P.V. (2021). Current methodologies to refine bioavailability, delivery, and therapeutic efficacy of plant flavonoids in cancer treatment. J. Nutr. Biochem..

[60] Shi G.J., Li Y., Cao Q.H., Wu H.X., Tang X.Y., Gao X.H., Yu J.Q., Chen Z., Yang Y. (2019). In vitro and in vivo evidence that quercetin protects against diabetes and its complications: A systematic review of the literature. Biomed. Pharmacother..

[61] Tang S.M., Deng X.T., Zhou J., Li Q.P., Ge X.X., Miao L. (2020). Pharmacological basis and new insights of quercetin action in respect to its anti-cancer effects. Biomed. Pharmacother..

[62] Cai X., Fang Z., Dou J., Yu A., Zhai G. (2013). Bioavailability of quercetin: Problems and promises. Curr. Med. Chem..

[63] Walle U.K., Walle T. (2007). Bioavailable flavonoids: Cytochrome P450-mediated metabolism of methoxyflavones. Drug Metab. Dispos..

[64] Chen X., Li D., Hu Y., Jin M., Zhou L., Peng K., Zheng H. (2011). Simultaneous determination of 3,3′,4′,5,7-pentamethylquercetin and its possible metabolite 3,3′,4′,7-tetramethylquercetin in dog plasma by liquid chromatography-tandem mass spectrometry and its application to preclinical pharmacokinetic study. J. Chromatogr. B Anal. Technol. Biomed. Life Sci..

[65] Vinayak M., Maurya A.K. (2019). Quercetin Loaded Nanoparticles in Targeting Cancer: Recent Development. Anti-Cancer Agents Med. Chem..

[66] Das S.S., Bharadwaj P., Bilal M., Barani M., Rahdar A., Taboada P., Bungau S., Kyzas G.Z. (2020). Stimuli-Responsive Polymeric Nanocarriers for Drug Delivery, Imaging, and Theragnosis. Polymers.

[67] Ghafelehbashi R., Tavakkoli Yaraki M., Heidarpoor Saremi L., Lajevardi A., Haratian M., Astinchap B., Rashidi A.M., Moradian R. (2020). A pH-responsive citric-acid/alpha-cyclodextrin-functionalized Fe_3_O_4_ nanoparticles as a nanocarrier for quercetin: An experimental and DFT study. Mater. Sci. Eng. C Mater. Biol. Appl..

