# Supplementary material for: Pentamethylquercetin Regulates Lipid Metabolism by Modulating Skeletal Muscle-Adipose Tissue Crosstalk in Obese Mice
Source: Pharmaceutics. 2022 May 29;14(6):1159. doi: 10.3390/pharmaceutics14061159 (PMC9227162; doi:10.3390/pharmaceutics14061159)
Supplement: Supplementary file 1 [file pharmaceutics-14-01159-s001.zip › pharmaceutics-1690083-supplementary.pdf]

Table S1 primer sequence

Gene name	Forward primer sequence (F)	Reverse primer sequence (R)
Ucp-1	AGGCTTCCAGTACCATTAGGT	CTGAGTGAGGCAAAGCTGATTT
Pgc-1α	GGAGCTGGATGGCTTGGGACAT	TTCGCAGGCTCATTGTTGTACTGGT
Cox-7a1	GCTCTGGTCCGGTCTTTTAGC	GTACTGGGAGGTCATTGTCGG
Cidea	TGACATTCATGGGATTGCAGAC	GGCCAGTTGTGATGACTAAGAC
Cd137	CGTGCAGAACTCCTGTGATAAC	GTCCACCTATGCTGGAGAAGG
Tmem26	ACCCTGTCATCCCACAGAG	TGTTTGGTGGAGTCCTAAGGTC
Tbx-1	GGCAGGCAGACGAATGTTC	TTGTCATCTACGGGCACAAAG
Fndc5	ATGAAGGAGATGGGGAGGAA	GCGGCAGAAGAGAGCTATAACA
Gapdh	GACAAAATGGTGAAGGTCGGTG	TGATGTTAGTGGGGTCTCGCTC

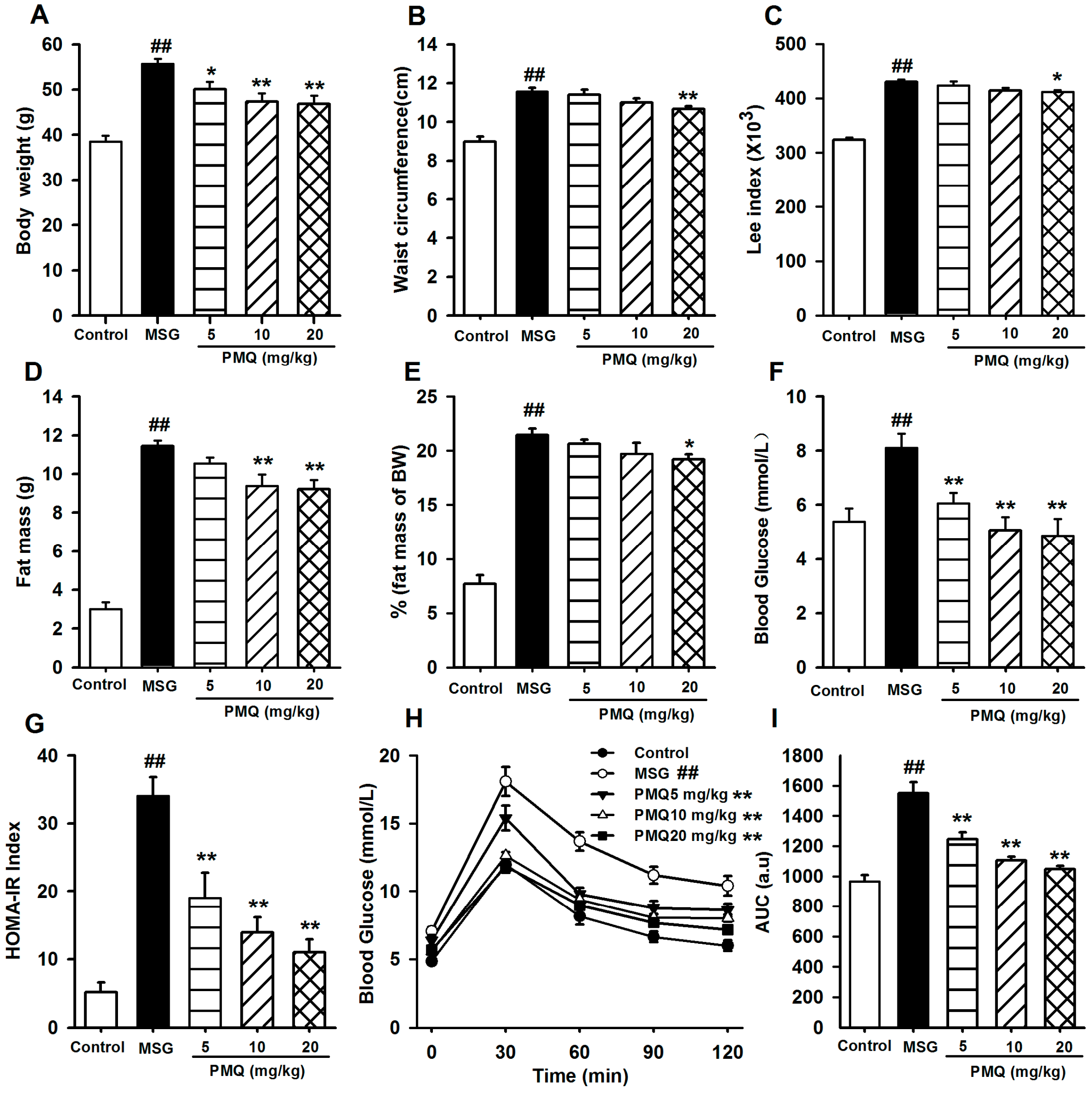

Figure S1 PMQ reduces obesity and improves insulin sensitivity. Vehicle or PMQ were administered orally to 5-week-old CD-1 or MSG-induced obese mice for 19 weeks, and body weight (A), waist circumference (B), Lee index (C), fat mass (D), fat mass percentage (E), blood glucose (F), HOMA-IR index (G), OGTT (H) and AUC (I) levels were determined. ##$P < 0.01$ vs control group; *$p < 0.05$, **$p < 0.01$ vs MSG group. Data are expressed as means ± SEM, n = 8-10.

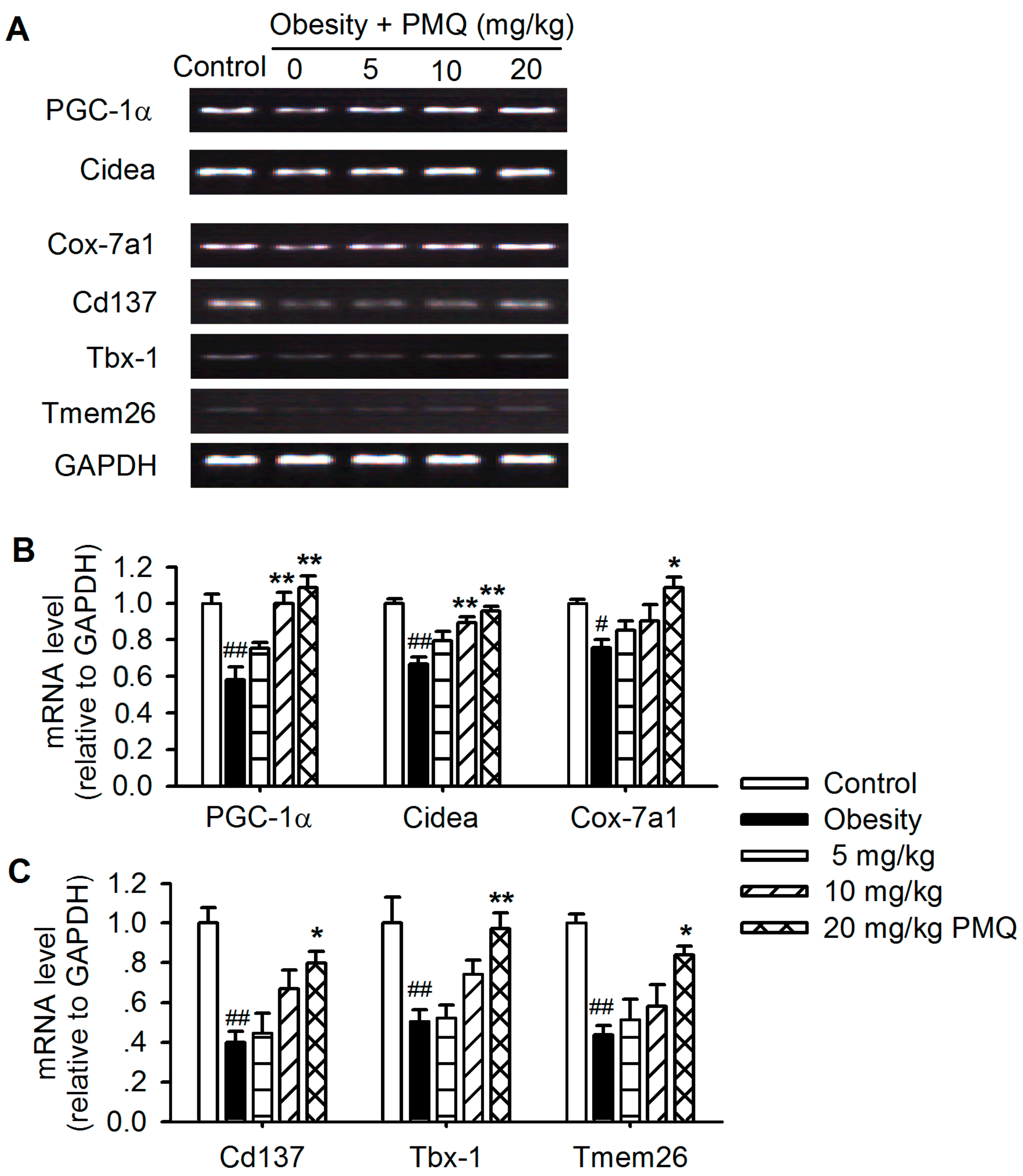

Figure S2. PMQ promotes the browning of inguinal WAT in MSG mice. (A) Representative PCR bands of brown-fat specific genes and beige cell markers in the inguinal depots. Each mRNA measurement was normalized to GAPDH (B, C). #$p < 0.05$, ##$p < 0.01$ vs control group; *$p < 0.05$, **$p < 0.01$ vs MSG group. Data are expressed as means ± SEM, $n = 4$.

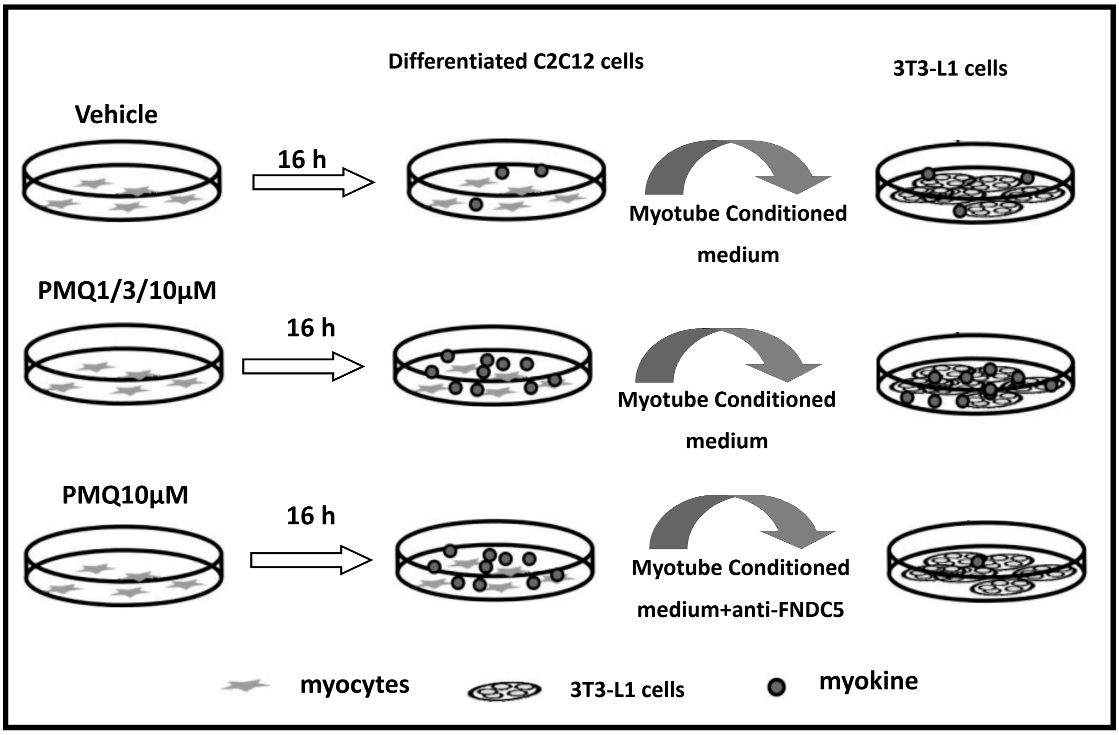

Figure S3. Experimental scheme for myotube-conditioned media treatment on 3T3-L1 adipocytes. Differentiated C2C12 cells were incubated with PMQ (1, 3, 10 μM) or vehicle for 16 h. Then the 24 h myotube-conditioned media were collected and filtered. Thereafter, the conditioned media were transferred to differentiation medium of 3T3-L1 adipocytes for 8 days until adipocytes matured.

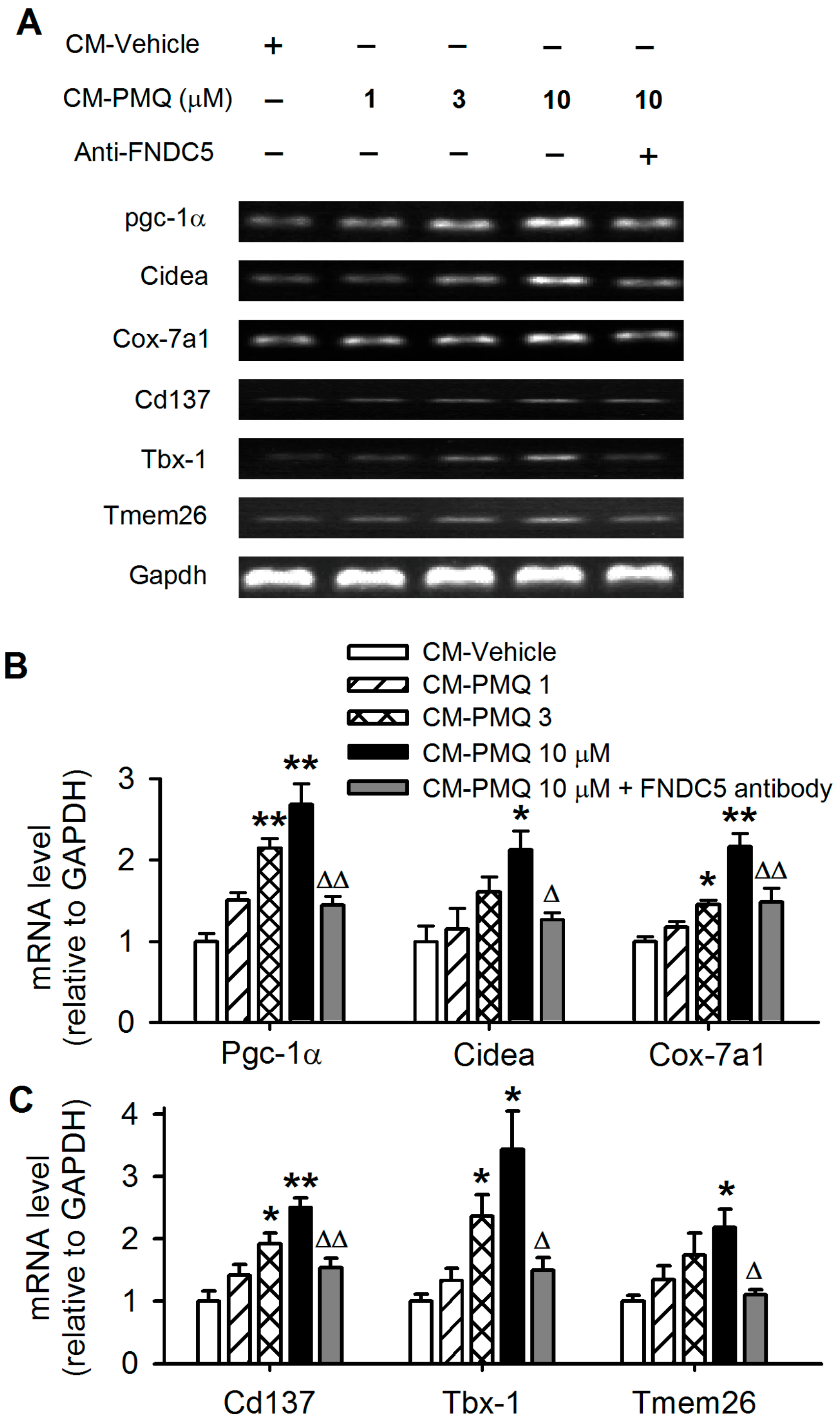

Figure S4. Conditioned media from PMQ-treated myotubes induce brown-like transition in 3T3-L1 cells. 3T3-L1 cells were treated with CM-vehicle, CM-PMQ (1, 3, 10) and CM-PMQ10 plus FNDC5 antibody (anti-FNDC5). (A) Representative PCR bands of brown-fat specific genes and beige cell markers in the inguinal depots. Each mRNA measurement was normalized to GAPDH (B, C). $n = 3$. *$p < 0.05$, **$p < 0.01$ vs MCM-vehicle; $\Delta P < 0.05$, $\Delta\Delta P < 0.01$ vs MCM-PMQ10. Data are expressed as means ± SEM.
